# Advancing Colorectal Cancer Detection With Blood-Based Tests: Qualitative Study and Discrete Choice Experiment to Elicit Population Preferences

**DOI:** 10.2196/53200

**Published:** 2024-07-17

**Authors:** Clarence Ong, Alex R Cook, Ker-Kan Tan, Yi Wang

**Affiliations:** 1 Saw Swee Hock School of Public Health National University of Singapore and National University Health System Singapore Singapore; 2 Department of Surgery Yong Loo Lin School of Medicine National University of Singapore Singapore Singapore

**Keywords:** blood-based test, colorectal cancer screening, mixed methods research, discrete choice experiment

## Abstract

**Background:**

Colorectal cancer (CRC) is the second most deadly form of cancer, inducing an estimated 1.9 million incidence cases and 0.9 million deaths worldwide in 2020. Despite the availability of screening tests, their uptake remains suboptimal. However, blood-based tests that look for signs of cancer-specific markers in the body are increasingly available as an alternative for more invasive tests for cancer. Compared with existing tests, the benefits of blood-based tests for CRC include not needing pretest preparation, stool handling, and dietary or medication restrictions.

**Objective:**

This study aims to explore the population’s preferences for CRC screening tests, with a focus on blood-based tests, and investigate the factors influencing test uptake.

**Methods:**

We used a mixed methods approach, combining semistructured interviews and a discrete choice experiment (DCE) survey. Interviews were analyzed using thematic analysis to identify salient attributes for CRC screening tests. These attributes informed the design of the DCE survey. The DCE data were analyzed using mixed logit and mixed-mixed multinomial logit models.

**Results:**

Qualitative findings from 30 participants revealed that participants preferred blood-based tests due to their perceived low risk, minimal pain, and ease of sample collection. However, concerns about the test’s lower accuracy were also expressed. The DCE survey was completed by 1189 participants. In the mixed logit model, participants demonstrated a stronger preference for blood-based tests over a 2-day stool-based test. The mixed-mixed multinomial logit model identified 2 classes, strong supporters and weak supporters, for CRC screening. Weak supporters, but not strong supporters, had a higher preference for blood-based tests. Women, ethnic Chinese, and people aged 40 to 60 years were more likely to be weak supporters. Both models highlighted the high influence of cost and test sensitivity on participants’ preferences. Transitioning from a 2-day stool-based test to a blood-based test, assuming a national screening program at a base price of Singapore $5 (US $3.75), was estimated to have the potential to increase the relative uptake by 5.9% (95% CI 3.6%-8.2%).

**Conclusions:**

These findings contribute to our understanding of CRC screening preferences and provide insights into the factors driving test uptake. This study highlights the perceived advantages of blood-based tests and identifies areas of concern regarding their accuracy. Further research is needed to determine the actual increase in uptake rate when blood-based tests are made available.

## Introduction

### Background

Colorectal cancer (CRC) is the second most deadly form of cancer, inducing an estimated 1.9 million incidence cases and 0.9 million deaths worldwide in 2020 [1]. Regular screening for CRC, through methods such as colonoscopy and fecal immunochemical testing (FIT), helps detect CRC earlier, reduces the incidence and mortality of CRC, and brings about cost savings compared with not undergoing any screening [2]. Despite the availability of screening as a preventive intervention for the early detection of CRC, screening rates are suboptimal, even within high-income countries [3-5]. Factors that hamper screening include not just individual characteristics but characteristics of the screening tests as well [6].

The emergence of blood-based, early detection tests for cancer has the potential not only for detecting multiple cancers but also for improving patient compliance and acceptance [7]. The discovery of circulating and cell-free tumor DNA in the blood has ushered in new possibilities for the blood-based detection of CRC as well [8]. *Epi proColon—*a *SEPT9* DNA methylation assay—remains to be the only US Food and Drug Administration–approved test [9]. However, there are at least 5 other blood-based tests in various stages of development, with tests ranging from CRC-specific tests to multi-cancer early detection tests. Some candidate analytic targets include cell-free DNA, methylated circulating tumor DNA, and a combination of methylated DNA and proteins [10]. Compared with existing tests, the benefits of blood-based tests for CRC include not needing pretest preparation, stool handling, and dietary or medication restrictions [9].

Challenges to the implementation of blood-based tests for screening include lower specificity relative to one-time FIT [11] and inferior sensitivity compared with next-generation FIT-DNA tests [12]. As a result, blood-based tests for CRC are not recommended for the general population in the health guidelines of the United States, Europe, China, and Singapore [13-16]. While there exist several clinical disadvantages to blood-based tests, it may serve as an alternative for patients refusing screening by colonoscopy or patients self-excluded from stool-based tests due to bleeding conditions such as hemorrhoids radiation proctitis [17]. In fact, the *SEPT9* test was found to be more effective and cost-effective compared with no screening [18]. By making screening easier, blood-based tests have the potential to improve uptake if the benefits outweigh the downside of this screening modality [19]. Studies are required to understand how the population will make trade-offs between different procedures and their attributes.

In a randomized controlled trial (RCT) involving average-risk adults that offered blood-based tests and FIT in a clinical setting, higher screening participation rates were observed in the blood-based test arm [20]. The blood-based test was also found to be effective in increasing screening rates among medically underserved populations [19]. However, another RCT reported no statistically significant improvement in the uptake among the population familiar with FIT if a blood-based test was offered upfront as an option [21]. Conversely, studies offering the blood-based test as a rescue option for those who declined colonoscopy and stool-based tests showed an increase in participant rates [21-23].

### Objective

Building on the existing literature, at least 4 questions are deserving of further investigation. First, what is the population’s inclination toward blood-based tests if the accuracy of blood-based tests can be improved to satisfactory levels akin to the FIT-DNA test? This insight can help assess the potential value of further investment in the test and inform the design of a target product profile [24,25]. Second, what is the general population’s preference for using the blood-based test in routine CRC screening? Results from RCTs may not be generalizable to the general population given the differences in the characteristics between the study participants and the general population. Third, considering heterogeneous preferences for blood-based tests, can we profile the population based on their preferences? Such profiling efforts can inform the crafting of targeted screening programs to cater to the heterogeneous preferences across different groups. Fourth, many preference studies were done in Western countries and very few were done in Asia [26,27]. Cultural and social norms could influence decision-making and outcomes. Studies understanding the acceptance of blood-based CRC tests in Asia are needed.

Acknowledging these gaps, we have designed a mixed methods study to delve into the population’s preference for blood-based testing modalities in Singapore, a multiracial Asian society, and to understand their decision-making process when choosing between blood-based tests and other existing screening methods. A discrete choice experiment (DCE) was used to construct hypothetical scenarios (eg, higher accuracy for the blood-based test). Furthermore, we intend to undertake subgroup analyses to examine potential variations in the preference for blood-based tests within distinct segments of the population, as highlighted by the mixed results of the RCTs. Our investigation will also delve into whether specific screening methods, such as the blood-based test, might positively impact participation rates, particularly in subpopulations identified with lower anticipated adherence based on prevailing screening recommendations.

## Methods

This is a mixed methods study with interviews and a survey that incorporated a DCE. The methods for the qualitative and quantitative components will first be outlined, and subsequently, the qualitative and quantitative results will be presented.

### Ethical Considerations

The study was approved by the National Healthcare Group Domain Specific Review Board (2021/00753) before data collection. Participants of the DCE were from a web-based cohort, and their participation in research was approved by the National University of Singapore (NUS) institutional review board (NUS-IRB: H-18-011).

### Qualitative Component

#### Participant and Sampling

The recruitment and interview of participants for the qualitative component took place between December 2021 and March 2022. Convenience sampling was undertaken to include the Singapore population aged ≥40 years with varied engagement with CRC screening services. Recruitment was conducted via the NUS social media platforms and its email blast services, as well as other participant recruitment channels and word of mouth. Interested potential participants contacted the researchers, who verified their eligibility before taking informed consent.

#### Conducting the Interview

The interviews took place either on the web via a videoconferencing application or in a quiet room within the NUS that was convenient for recording. The interviews adopted a semistructured format using a topic guide. Each interview lasted approximately 30 to 45 minutes and was conducted in English.

#### Analysis of Interviews

The interviews were transcribed verbatim, and the data were analyzed using thematic analysis. A preliminary codebook with emerging themes of relevance from the first 5 transcripts was developed upon full familiarization of the transcripts. A deductive and semantic approach was used in the clustering of codes into metacodes and categories of interest. The coding framework was subsequently applied to the remaining transcripts. The identified themes were also further reviewed to ensure their usefulness and accuracy in representing the data.

### Quantitative Component

#### Discrete Choice Experiment

DCEs have increasingly become a popular method for investigating and eliciting patient and population preferences for health care [28]. The method is based on consumer choice theory [29], which posits that respondents make choices between hypothetical products or scenarios comprising of ≥2 alternatives based on the importance they place on the characteristics of these attributes. In a DCE, a product or scenario is described with a fixed number of attributes with varying combinations of levels. Per this paradigm, in choosing the ideal product or scenario, the respondent evaluates the overall desirability of the alternatives and makes trade-offs among the attributes. From the respondents’ choice, their preferences are indirectly revealed, determining the attributes that drive the respondents’ preferences as well as the way variations of the attributes and levels may affect the respective preferences [30].

#### Selection of Attributes and Levels

The selection of attributes and levels must be relevant to the policy process and the study population, while being consistent with the random utility theoretical foundation of DCEs [31]. An initial set of attributes and levels for the DCE was based on a scoping review of the existing literature, which yielded 13 attributes. During the aforementioned qualitative interviews, participants were asked to rank 3 attributes that they valued the most, and the weighted preferences of all participants helped shortlist the final attribute list for the DCE. Following that, a quantitative survey with eligible health care professionals (n=11), who had at least 1-year working experience with patients with CRC or RC screening, was conducted to ensure the validity of the selected attributes and their corresponding levels. After these iterative processes, six attributes were identified and ultimately used in the DCE: (1) procedure, (2) pain level, (3) sensitivity, (4) recommendation, (5) out-of-pocket cost, and (6) risk of test. Each attribute was assigned various levels based on the best information available. The blood-based test was one level of the procedure attribute.

To optimize the choice sets, a pilot study was conducted with 12 participants. Adjustments were then made to the text for the attributes and levels to improve clarity for the participants. The final set of 6 attributes and levels is presented in Textbox 1. The total number of appearances and selections for each attribute and level may be found in Multimedia Appendix 1.

**Attributes and levels in the discrete choice experiment.**

- **Procedure**
  Colonoscopy
  Computed tomography colonography
  Stool-based (2 days)
  Stool-based (1 day)
  Blood-based
- **Pain level**
  No pain
  Mild pain
- **Sensitivity**
  100%
  95%
  80%
  60%
- **Recommendation**
  Health Promotion Board
  Doctors
  Family or friends
  Neither
- **Cost**
  Singapore $0
  Singapore $5 (approximately US $3.75)
  Singapore $30 (approximately US $22.50)
  Singapore $400 (approximately US $300)
  Singapore $1000 (approximately US $750)
- **Risk of test**
  No risk
  1% risk of adverse event

#### Experimental Design

The DCE questionnaire was designed using Sawtooth Lighthouse Studio (version 9.13.2), and a 2-stage design was used. For each task, participants first selected the preferred choice from 2 test profiles and were then asked to choose between taking the test or opting out of it in real life. Correspondingly, the parameter labeled “Opt-Out” represents the utility associated with declining the preferred test in the first stage. A negative value thus indicates a participant’s preference to undergo the screening test. The questionnaire was designed using the random task generation method provided by Sawtooth. The DCE included a total of 20 blocks with 10 choice sets each. Each study participant saw 1 block of choice sets, consisting of 10 choice sets, from the 20 blocks. To test for internal validity, 1 fixed choice set offering 2 alternatives is common across all blocks, of which one is intended to be strictly dominant over the other.

**Figure 1:**
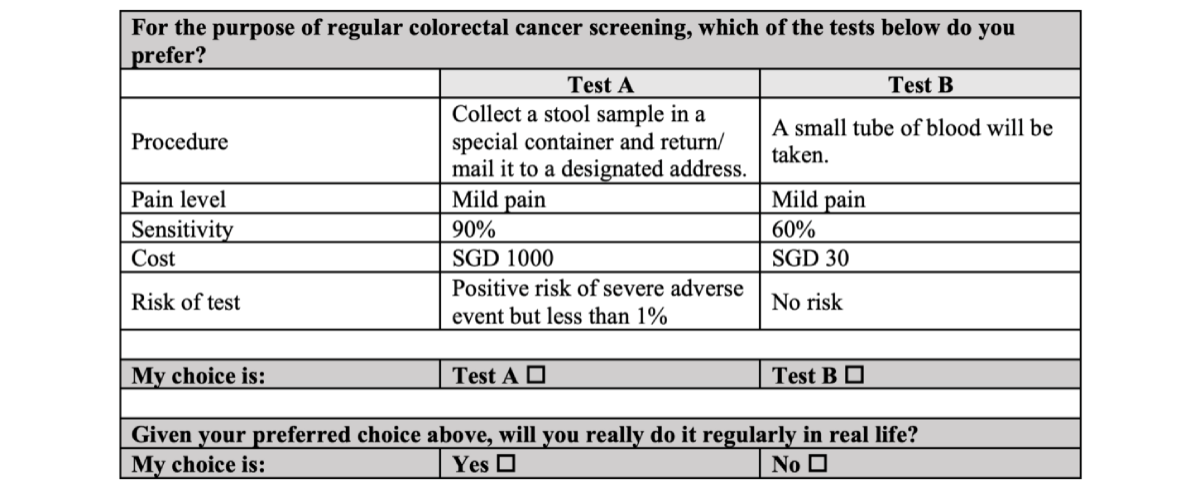
An example of a discrete choice experiment choice task. SDG: Singapore Dollar (SGD 1=approximately US $0.75).

The levels of cost were selected to reflect the costs of different procedures in reality. Participants in qualitative interviews also demonstrated a similar perception regarding the cost of the tests. Certain within-concept prohibitions were also specified to provide combinations of attributes that were realistic. This included prohibiting high out-of-pocket payment costs and the presence of mild pain for stool-based tests. However, we allowed the blood-based test to appear together with a higher cost given that commercial companies may set higher prices [32]. The coverage matrix of the DCE design was examined using Sawtooth Lighthouse Studio to ensure all the parameters can be estimated. In addition, considering the large range of costs, we treated cost as discrete variables rather than a continuous variable in our analysis. An example of a DCE choice task is presented in Figure 1.

Participants reported any familial history of CRC and if they had attended any type of CRC screening in the past. Various sociodemographic information was also collected. All variables were coded as categorical variables, and some variables were subsequently recoded as binary or ternary variables to form meaningful subgroups for analyses. Psychosocial inventories—the Zimbardo Time Perspective Inventory [33], the Intolerance of Uncertainty Scale [34], and the Duke UNC Functional Social Support Questionnaire [35]—were also included to measure participants’ degree of present orientation, intolerance of uncertainty, and social support, respectively.

#### Participant and Sampling

This study was conducted as a web-based survey hosted on REDCap from May 19, 2022, to May 28, 2022. The target population for this study was Singapore citizens and permanent residents aged ≥40 years. The survey was sent to the participants from Singapore Population Health Studies web-based panel. This web-based panel consists of a cohort that is broadly representative of the general Singapore population. Participants gave their implicit consent by participating in the survey and were reimbursed with Singapore $10 (approximately US $7.50) for every successful completion of the survey.

In calculating the minimum sample size, we made use of the proposed formula of n>500*c/(t*a) by Johnson and Orme [36], where 500 is a fixed variable, *c* demotes the largest number of levels for a certain attribute, *a* indicates the number of DCE choice sets per block of questionnaire, and *t* refers to the number of alternatives per DCE choice set (excluding “Opt-out”). Accordingly, the sample size required for this study should be >125 participants (500*5/(2*10)=125). In addition, Lancsar and Louviere [30] suggested 20 responses per block or questionnaire, which led to a minimum sample size of 400. Considering that the DCE may be relatively difficult to answer, we expected a relatively high nonresponse rate of 20% to 30%. Hence, we set the target sample size to be 600. We subsequently tested the sample size using simulation functions with Sawtooth Lighthouse Studio, which deemed the sample size of 600 as sufficient. Owing to the overwhelming response, our final sample size of 1189 participants not only meets the minimum required sample size but also allows for the possibility of conducting flexible subgroup analyses.

#### Statistical Analysis

A total of 2 models were tested: a mixed logit (MXL) model and a mixed-mixed multinomial logit (MMML) model [37]. The MXL model was selected to account for the correlation introduced by the repeated observations from each participant and to relax the assumptions of independence from irrelevant alternatives [38]. This model assumes that the choices made by the same participant are correlated, and preference heterogeneity exists across the population sample. The interpretation of the mean preference weights is made in relation to the chosen base level, and the SD of each level indicates the variability in the mean preference weights. The MMML model was also selected as it incorporates both MXL and the latent class logit model. Unlike latent class logit where a homogeneous fixed preference is assumed within each latent class, a distribution of random coefficients is specified in the MMML model. Within each class, preference weights and the average probability of each demographic within each class can be derived. The number of classes was chosen by examining the Bayesian information criterion.

In addition, the conditional relative importance (CRI) for a given attribute, defined as the difference between the highest preference weight of the attribute level and the lowest preference weight of the attribute level, was reported. A higher CRI indicates the attribute is more important in designing the CRC screening program. Profile-based normalization was then applied to normalize the sum of CRI of all the attributes to 1.

A left-specific constant was included in each regression, with a statistically significant coefficient indicating a left-right bias in the study [39]. Participants in DCE may take shortcuts and use simplifying heuristics when answering DCE questions, which can introduce an unintended source of variability in the data. When using reading order heuristic, study participants may tend to choose the choice on one side [40]. Incorporating a variable indicating the position of the choice in the regression can disentangle the associated bias [41,42].

All levels were coded as dummy variables. Reference levels were selected based on the current recommendation for CRC screening for the average-risk adult population. However, we intentionally designated “No Recommendation” as the reference level for the “Recommendation” attribute to investigate the nuanced preferences arising from diverse recommendations made by different individuals. Continuous psychological variables were demedian by subtracting the median value from each data point, resulting in a centered distribution. As for the classification of the continuous sociodemographic variables, age bands were set according to the definition of senior citizen locally, which is at the age of ≥60 years. Household income was grouped to ensure a sufficient sample size in both groups.

Statistical significance was set at *P*<.05. All quantitative data analyses were carried out using the statistical software R (version 4.2.1; The R Foundation) [43].

## Results

### Qualitative Component

#### Sample Characteristics

A total of 30 participants completed the interview. A summary of their sociodemographic characteristics is presented in Multimedia Appendix 2. Of these participants, 14 had undergone a colonoscopy, 20 had taken a stool test, and 6 had taken a blood-based test. A total of 5 participants had not undergone any screening for CRC. Five main themes that elucidated the participants’ motivations and important attributes of CRC screening were identified: (1) accuracy, (2) cost, (3) perceived risk and pain, (4) convenience of the test, and (5) the method of sample collection.

#### Accuracy

When it came to blood-based tests, many participants were uncomfortable with its inferior accuracy relative to other tests. They saw it as “pointless” due to its possibility of giving rise to many false positives and negatives:

...half the chance of being accurate, then why waste my blood?

P6, female

If [accuracy is] too low, then it defeats the purpose already...you want to know whether you have cancer or not, you see.

P24, female

For participants who felt that they had no symptoms, the level of accuracy of the stool-based tests provided sufficient assurance that they felt a colonoscopy was unnecessary. Many cited an approach of escalation—to undergo a colonoscopy only in the event of a positive stool-based test:

I’m not in the high-risk category...I’ll go for the FIT first, and then based on [the results], I’ll go for the colonoscopy.

P22, female

If I have concerns, colonoscopy will be the best. But if it’s more like a routine check, then I think stool-based would be better.

P25, male

Participants who had undergone invasive tests such as colonoscopy either (1) were diagnosed with hemorrhoids or (2) had discovered blood in their stools. They had opted for the colonoscopy under their physician’s or friend’s advice, seeing that a colonoscopy was a more “comprehensive” or “complete” test. Many felt that the relatively higher accuracy of the colonoscopy provided them greater ease of mind:

[Colonoscopy] gives a more accurate reading, because you’re able to see...what’s inside.

P19, female

[Colonoscopy] is not so comfortable, but it’s comfortable to the mind.

P30, male

#### Perceived Risk and Pain

The stool-based and blood-based tests were more favorable to most participants as the process was “simple” and “pain-free.” Furthermore, many who had undergone a blood-based test did so as part of their annual comprehensive health check-up and saw little extra risk or pain in doing it:

It’s less traumatic to the patient. That kind of [needle] pain is bearable...no issue.

P1, female

If it’s part of the blood works, might as well, right? Since they are already drawing blood? I don’t mind testing for [colorectal cancer] as well.

P17, female

Many of these participants who had no prior experience undergoing colonoscopy were more likely to express fear of the risk of colon perforation from the procedure. Due to its invasive nature, many were also “scared” and “uncomfortable” with the pain the procedure might induce:

I don’t know how big the scope is...how difficult is it to insert? Will it damage anything permanently?

P11, male

The sort that comes with pain...[I] may get cold feet.

P17, female

Interestingly, most participants who had undergone colonoscopy had little qualms about the risk and pain of the procedure. Instead, many expressed difficulties adhering to the bowel preparation instead:

The agony part was the bowel preparation. I can’t finish the 4 litres...I gave up at 2 litres.

P14, male

#### Method of Sample Collection

While being relatively easy to conduct, some participants shared reservations about the collection of stool samples. They saw the process as “dirty,” “disgusting,” or “troublesome,” especially when 2 separate stool samples were required. While some complained about the uncomfortable experience of stool collection, some expressed personal concerns about improper collection:

...because you have to do it on your own, especially when you have to dig the stool, I’m not sure whether we are doing correctly or not.

P28, male

Nevertheless, participants who have done it across many years expressed little of such concerns, seeing it as a routine exercise that was necessary:

You’ve done it once and then you make it an annual exercise...it’s not a big deal.

P10, female

#### Convenience of Test

As colonoscopies and blood-based tests require medical professionals to perform them, some participants felt that the need to arrange a doctor’s appointment was time-consuming. This was especially true for colonoscopies, where a referral from a primary care physician is required to receive a subsidized rate for the colonoscopy:

...you have to go to the [primary care] polyclinic and then get a referral, see the specialist and then wait for the appointment...you know, so it’s a bit more cumbersome.

P25, male

However, participants shared that stool-based tests were relatively more accessible, with kits being easily obtained at pharmacies or mailed to them on request. Even when returning stool samples, some participants shared that mailing services to the laboratory were available, which saved them the shame and hassle of dropping them off at a clinic:

[It’s] kind of a deterrent because you have a book an appointment...compared to FIT test, you can just drop by any of the pharmacies...it’s a lot more convenient.

P20, female

#### Cost

Many participants were aware of the stark difference in cost between a colonoscopy and a stool-based or blood-based test. While many participants, especially those younger and working, had employer and private insurances to subsidize a colonoscopy procedure, they highlighted that out-of-pocket cost was still substantially higher. Many cited that a higher willingness to pay must come in tandem with either higher accuracy and lower frequency of testing:

If the colonoscopy is 70-80% [accurate], and the other tests are also 70-80% [accurate], of course I will choose the simple one. No point to go for a colonoscopy...pay so much, go through the hassle...

P24, female

[If] you do this scope, one time, last you for 10 years, [because them you] don’t have to collect stool sample at the next medical check-up.

P29, male

### Quantitative Component

#### Sample Statistics

A total of 1189 participants completed the study. Of these, 127 (10.7%) participants did not have complete sociodemographic information, while 44 (3.7%) participants failed the fixed choice task. This led to 168 (14.1%) participants dropping out, leaving a sample of 1021 participants for analysis. The demographic characteristics of all participants are presented in the Multimedia Appendix 2.

#### MXL Model

The main results of the MXL model are presented in Table 1.

All mean coefficients were significant at *P*<.05. On average, participants exhibited a higher preference for blood-based tests relative to a 2-day stool-based test (coefficient=0.40, 95% CI 0.24-0.55). Participants also exhibited a higher preference for a 1-day stool-based test relative to a 2-day stool-based test (coefficient=0.27, 95% CI 0.10-0.45). The preference for these 2 procedures were however not statistically different from each other (coefficient=0.12, 95% CI –0.04 to 0.29).

**Figure 2:**
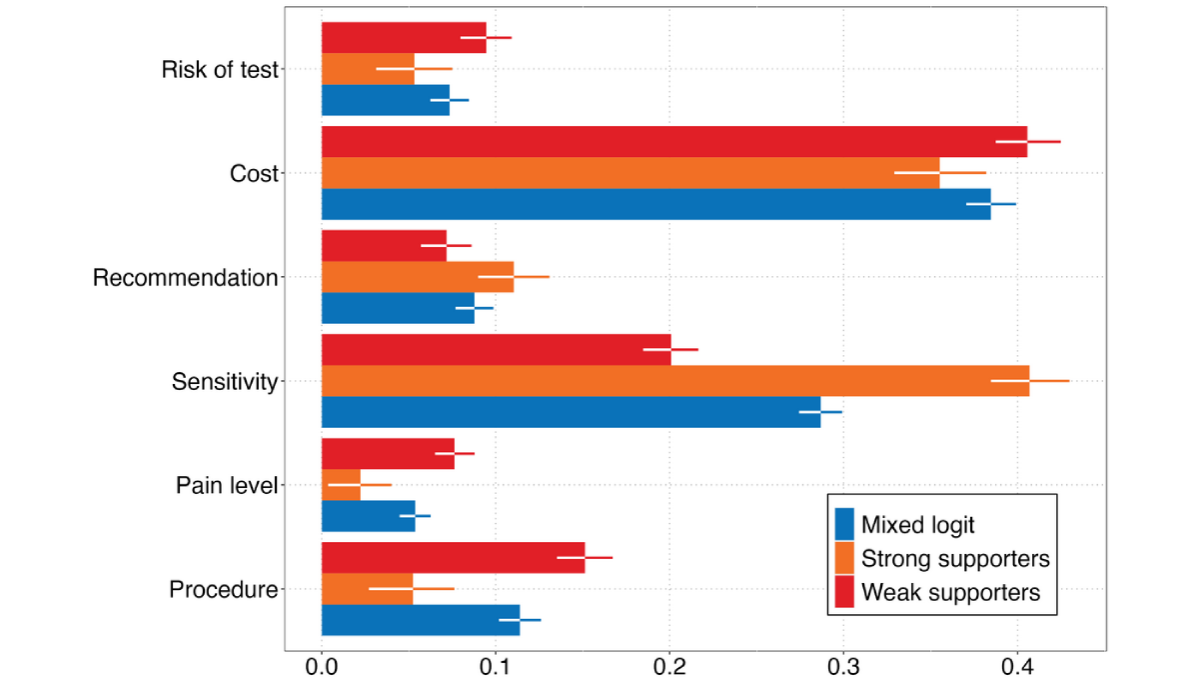
Profile-based normalized conditional relative importance. Strong supporters and weak supporters are from mixed-mixed multinomial logit model. Numbers on the x-axis represent the relative weight of each attribute in each model. The relative weights of each attribute within a model sums up to 1. A higher value means the attribute is more important in decision-making.

The profile-based normalized CRI of the 6 attributes based on the MXL model is presented in Figure 2. Ranking the attributes, participants were most concerned with the cost and sensitivity of the screening test. This is followed by the procedure type, the risk level, the pain level, and ultimately the recommendation received for the screening test.

**Table 1:** Mixed logit analysis^a,b^.

			Mean					SD		
			Coefficient (95% CI^b^)		*P* value		Coefficient (95% CI)			*P* value
Left			0.13 (0.06 to 0.12)		<.001		—^c^			—
Opt-out			−1.74 (−1.93 to −1.55)		<.001		3.86 (3.67 to 4.05)			<.001
**Procedure**										
	Colonoscopy	−0.73 (−0.90 to −0.57)		<.001		0.98 (0.85 to 1.12)			<.001	
	CT^d^ colonography	−0.75 (−0.91 to −0.60)		<.001		0.65 (0.51 to 0.80)			<.001	
	Stool-based (2 days)	0.00 (Reference)		—		0.00 (Reference)			—	
	Stool-based (1 day)	0.27 (0.10 to 0.45)		<.001		0.93 (0.71 to 1.16)			<.001	
	Blood-based	0.40 (0.24 to 0.55)		<.001		0.91 (0.76 to 1.05)			<.001	
**Pain level**										
	No pain	0.00 (Reference)		—		0.00 (Reference)			—	
	Mild pain	−0.54 (−0.64 to −0.45)		<.001		0.26 (0.11 to 0.40)			<.001	
**Sensitivity (%)**										
	100	1.63 (1.52 to 1.75)		<.001		0.09 (−0.05 to 0.24)			0.19	
	95	0.70 (0.59 to 0.82)		<.001		0.02 (−0.13 to 0.18)			0.79	
	80	0.00 (Reference)		—		0.00 (Reference)			—	
	60	−1.26 (−1.43 to −1.08)		<.001		1.74 (1.53 to 1.95)			<.001	
**Recommendation**										
	Health Promotion Board	0.88 (0.77 to 1.00)		<.001		0.11 (−0.04 to 0.25)			.16	
	Doctors	0.68 (0.56 to 0.80)		<.001		0.01 (−0.14 to 0.17)			.86	
	Family or friends	0.35 (0.23 to 0.48)		<.001		0.21 (0.05 to 0.36)			.008	
	Neither	0.00 (Reference)		—		0.00 (Reference)			—	
**Cost (Singapore $^e^)**										
	0	0.00 (Reference)		—		0.00 (Reference)			—	
	5	−0.35 (−0.50 to −0.19)		<.001		0.38 (0.16 to 0.59)			<.001	
	30	−0.81 (−0.93 to −0.69)		<.001		0.08 (−0.09 to 0.25)			.35	
	400	−2.39 (−2.53 to −2.25)		<.001		0.46 (0.30 to 0.62)			<.001	
	1000	−3.88 (−4.07 to −3.68)		<.001		1.53 (1.33 to 1.73)			<.001	
**Risk of test**										
	No risk	0.00 (Reference)		—		0.00 (Reference)			—	
	1% risk of adverse event	−0.74 (−0.86 to −0.62)		<.001		0.05 (−0.11 to 0.21)			.53	

^a^Log-likelihood: −8353, Akaike information criteria: 16,777, and Bayesian information criteria: 17,054.

^b^Mean refers to the population mean. SD measures the individual preference heterogeneity. A significant value means that the preference for the corresponding level is heterogeneous at the individual levels.

^c^Not applicable.

^d^CT: computed tomography.

^e^Singapore $1=approximately US $0.75.

#### MMML Model: Preference Analysis

The results from the MXL model suggested preference heterogeneity across the participants, with the SDs of several preferences being statistically significant. Thus, we ran an MMML model while also estimating the posterior class membership probabilities. A 2-class MMML model was the most appropriate based on the BIC. The classes were labeled post hoc as (1) strong supporters and (2) weak supporters, based on the coefficient value for “None.” A more negative coefficient value means people are more willing to take the screening test in real life. The class shares for strong supporters and weak supporters are 38.09% (n=339) and 61.91% (n=632), respectively. The main results of the MMML model are presented in Table 2. The full table with SD is presented in Multimedia Appendix 3.

Strong supporters did not exhibit a higher preference for blood-based tests relative to a 2-day stool-based test. However, weak supporters had a higher preference for blood-based tests compared with a 2-day stool-based test (coefficient=0.66, 95% CI 0.44-0.88). Similar to the results in the MXL model, strong and weak supporters were most concerned with the cost and sensitivity of the screening test. Weak supporters were more likely than strong supporters to be concerned with the procedure, pain level, and risk of test. Compared with an existing national screening program that is 2-day stool-based, has no pain, 80% sensitivity, recommended by the government’s Health Promotion Board, costs Singapore $5, and has no risk, the relative uptake rate of a blood-based screening test with all else constant will increase by 0.2% (95% CI −1.2% to 1.6%) for strong supporters and increase by 5.9% (95% CI 3.6% to 8.2%) for weak supporters.

**Table 2:** Mixed-mixed multinomial logit analysis^a^.

					Class 1: Strong supporters							Class 2: Weak supporters			
					Coefficient (95% CI)			*P* value			Coefficient (95% CI)				*P* value
**Mean coefficient**															
	Left			0.28 (0.15 to 0.42)			<.001			0.08 (−0.02 to 0.18)				.13	
	None			−5.13 (−5.79 to −4.46)			<.001			−0.86 (−1.10 to −0.61)				<.001	
	**Procedure**														
		Colonoscopy	−0.35 (−0.71 to 0.02)			.07			−1.05 (−1.28 to −0.81)				<.001		
		CT^b^ colonography	−0.53 (−0.87 to −0.18)			<.001			−1.01 (−1.23 to −0.78)				<.001		
		Stool-based (2 days)	0.00 (Reference)			—^c^			0.00 (Reference)				—		
		Stool-based (1 day)	−0.20 (−0.57 to 0.16)			.27			0.63 (0.39 to 0.87)				<.001		
		Blood-based	0.06 (−0.27 to 0.49)			.70			0.66 (0.44 to 0.88)				<.001		
	**Pain level**														
		No pain	0.00 (Reference)			—			0.00 (Reference)				—		
		Mild pain	−0.25 (−0.46 to −0.04)			.02			−0.84 (−0.98 to −0.70)				<.001		
	**Sensitivity (%)**														
		100	2.92 (2.61 to 3.24)			<.001			1.25 (1.10 to 1.41)				<.001		
		95	1.58 (1.29 to 1.87)			<.001			0.42 (0.25 to 0.58)				<.001		
		80	0.00 (Reference)			—			0.00 (Reference)				—		
		60	−1.65 (−2.04 to −1.27)			<.001			−0.96 (−1.19 to −0.72)				<.001		
	**Recommendation**														
		Health Promotion Board	1.24 (0.99 to 1.49)			<.001			0.79 (0.62 to 0.96)				<.001		
		Doctors	0.89 (0.64 to 1.14)			<.001			0.67 (0.50 to 0.83)				<.001		
		Family or friends	0.17 (−0.08 to 0.43)			.18			0.40 (0.22 to 0.58)				<.001		
		Neither	0.00 (Reference)			—			0.00 (Reference)				—		
	**Cost (Singapore $^d^)**														
		0	0.00 (Reference)			—			0.00 (Reference)				—		
		5	−0.67 (−1.02 to −0.32)			<.001			−0.33 (−0.55 to −0.11)				<.001		
		30	−0.97 (−1.24 to −0.70)			<.001			−0.83 (−0.99 to −0.66)				<.001		
		400	−2.24 (−2.55 to −1.94)			<.001			−2.93 (−3.14 to −2.72)				<.001		
		1000	−4.00 (−4.41 to −3.59)			<.001			−4.46 (−4.75 to −4.18)				<.001		
	**Risk of test**														
		No risk	0.00 (Reference)			—			0.00 (Reference)				—		
		1% risk of adverse event	−0.60 (−0.85 to −0.35)			<.001			−1.04 (−1.21 to −0.87)				<.001		
**Class membership**															
	**Sex**														
		Female	—			—			0.19 (0.11 to 0.27)				<.001		
		Male	—			—			0.00 (Reference)				—		
	**Ethnicity**														
		Chinese	—			—			0.52 (0.41 to 0.63)				<.001		
		Non-Chinese	—			—			0.00 (Reference)				—		
	**Age (years)**														
		40-60	—			—			0.00 (Reference)				—		
		≥61	—			—			−0.52 (−0.61 to −0.42)				<.001		
	**Household income level (Singapore $)**														
		High income (≥6000)	—			—			−0.13 (−0.22 to −0.05)				<.001		
		Lower income (≤5999)	—			—			0.00 (Reference)				—		
	**Marital status**														
		Married	—			—			−0.06 (−0.16 to 0.04)				.21		
		Single or divorced or widowed or separated	—			—			0.00 (Reference)				—		
	**Education level**														
		Primary and secondary	—			—			0.08 (−0.02 to 0.19)				.13		
		Preuniversity	—			—			0.00 (Reference)				—		
		University and above	—			—			0.02 (−0.22 to −0.05)				.61		
	**Housing type**														
		Public housing	—			—			0.00 (Reference)				—		
		Private housing	—			—			−0.47 (−0.60 to −0.35)				<.001		
	**Working status**														
		Currently working	—			—			−0.24 (−0.34 to −0.14)				<.001		
		Not working or retired or student	—			—			0.00 (Reference)				—		
	**Family history of CRC^e^**														
		Yes	—			—			−0.74 (−0.85 to −0.63)				<.001		
		No	—			—			0.00 (Reference)				—		
	**CRC screening history**														
		Yes	—			—			−0.52 (−0.60 to −0.44)				<.001		
		No	—			—			0.00 (Reference)				—		
	Perceived safety of test score			—			—			−0.16 (−0.17 to −0.14)				<.001	
	Social support score			—			—			−0.03 (−0.05 to −0.02)				<.001	
	Present orientation			—			—			−0.03 (−0.05 to −0.02)				<.001	
	Intolerance of uncertainty			—			—			−0.01 (−0.02 to −0.01)				<.001	

^a^Log-likelihood: −8041, Akaike information criteria: 16,257, Bayesian information criteria: 16,955.

^b^CT: computed tomography.

^c^Not applicable.

^d^Singapore $1=approximately US $0.75.

^e^CRC: colorectal cancer.

#### MMML Model: Analysis of Demographic Information

Weak supporters were more likely to be women, ethnic Chinese, and people aged 40 to 60 years. However, strong supporters were more likely to be working, have higher income levels, and live in private housing. Strong supporters were also more likely to have a family history of CRC and to have opted for CRC screening in the past. Compared with weak supporters, strong supporters were also found to have higher scores for the perceived safety of CRC screening tests, social support, present orientation, and intolerance of uncertainty.

## Discussion

### Principal Findings

For every screening program to be successful, it is important to identify patterns in the population’s choice for CRC screening test to encourage uptake. By using a sequential exploratory mixed methods design and using a web-based cohort that is designed to be representative of the general Singapore population, our study is able to identify salient attributes of screening among participants to inform our understanding of the population’s preference. Furthermore, controlling for some of the salient attributes in our DCE allows for a better interpretation of the preference of specific procedures, which is likely to encompass the value of convenience and method of sample collection that is otherwise not considered in the DCE.

Our DCE results suggest that most participants preferred a blood-based test over a 2-day stool-based test and colonoscopy after accounting for the other attributes (eg, sensitivity). A blood-based test was perceived to be pain free, with a method of sample collection that was relatively simpler, compared with a colonoscopy. This pattern of aversion to aspects of pain and risk that comes with colonoscopy is supported by several studies [44,45]. Furthermore, studies have also supported the convenience and ease of the blood-based tests as compared with colonoscopy, as no preparation is involved [44,46]. Stool-based tests were regarded as unpleasant and disgusting to some participants in line with studies from the United States [47], Australia [48], and Germany [49], wherein participants expressed a preference for blood over stool sampling. However, comparing a blood-based test with a 1-day stool-based test, the utility for a blood-based test was not statistically significantly higher. This suggests that the requirement of testing twice was a key contributor to the perceived inconvenience and disutility of a stool-based test.

One advantage of DCE is its ability to profile the population and understand the preference and estimate the uptake in different subpopulations. We profiled the population into 2 classes, the strong supporters for CRC screening who were indifferent between the 2-day stool-based test and blood-based test, and the weak supporters for CRC screening who preferred the blood-based test over the 2-day stool-based test. Our results suggest that the sensitivity of tests was a key consideration of participants. For example, a 2-day stool-based test with a sensitivity of 80% was preferred by both strong supporters and weak supporters compared with a blood-based test with a sensitivity of 60%. Unfortunately, the shortcoming of existing blood-based tests lies in the relatively inferior sensitivity and specificity [11,12], which was a concern for participants based on the qualitative interviews as well. In reality, while the procedure of blood-based tests itself is preferred, the low sensitivity and specificity rate is unlikely to appeal to the masses. However, if blood-based tests can achieve the same sensitivity as 2-day stool-based tests, by offering blood-based tests to weak supporters, there is a potential to increase the relative CRC screening uptake by approximately 6%. The impact is likely to be significant as weak supporters make up approximately 62% of the population. Hence, further investment in research and development to improve the accuracy of blood-based tests could be beneficial to society.

The weak supporters identified in the study were less likely to do CRC screening in real life, but showed a higher preference for blood-based tests. Several observable demographic factors were associated with being weak supporters, including being female, ethnic Chinese, and younger and having lower income. The government may thus use targeted information campaigns when blood-based tests become a feasible screening option. The convenience of the blood-based test needs to be highlighted to weak supporters. However, one concern identified from the interview was the need for health care providers to draw blood. Strategies and logistic arrangements to reduce waiting time for taking the blood-based test need to be designed. For strong supporters, relative to the testing procedure, they cared more about the sensitivity of the test compared with weak supporters. Blood-based tests itself are not attractive to them. Information campaigns to strong supporters should focus on better accuracy (eg, a blood-based test with high accuracy). Both strong supporters and weak supporters valued the recommendation from the Health Promotion Board [50] most, the governmental agency driving preventive care under the Ministry of Health in Singapore.

While DCEs can inform patients’ preferences on specific tests, a proper health technology assessment should be performed on whether blood-based tests are appropriate in each country. Unfortunately, cost-effectiveness studies on *SEPT9* were rarely included in systematic reviews of the cost-effectiveness of CRC screening strategies [51,52]. All found studies that included *SEPT9* as a screening strategy discovered that an annual *SEPT9* screening was more cost-effective than no screening [18,53,54]. A total of 2 studies that relied on test characteristics of the earlier version of *SEPT9* found that annual screening with FIT dominated the *SEPT9* strategy [18,54], meaning that using FIT provided superior outcomes at a lower cost compared with using *SEPT9*. However, a more recent study using test characteristics of the improved version of SEPT9 with higher sensitivity and specificity found that the strategy resulted in higher quality-adjusted life years gained, CRC cases adverted, and CRC deaths adverted compared with other screening strategies [53]. Nevertheless, the strategy of using *SEPT9* remained more costly than FIT as it resulted in a 63% higher referral for colonoscopy than FIT, increasing the cost by 26%. As a result, the strategy of FIT was still more cost-effective than *SEPT9*. However, these conclusions were based on perfect adherence of strategies. On the basis of our findings, the blood-based tests like *SEPT9* are more likely to have higher adherence than stool-based tests such as FIT if similar accuracy can be achieved. Indeed, 2 studies that considered imperfect uptake found that when the uptake rate of FIT fell below 70% relative to that of *SEPT9*, FIT was no longer more cost-effective than *SEPT9* [18,54]. Thus, the possibility of *SEPT9* being more cost-effective than FIT likely hinges on (1) an improved version of *SEPT9* with higher sensitivity and specificity and (2) a significantly higher uptake for *SEPT9* over FIT*.*

### Policy Implications

It is inevitable that the next frontier of cancer screening will be the adoption of blood-based tests [8]. Multiple such tests are being evaluated or studied currently [7]. Hence, it is no longer far-fetched that policy makers need to decide whether and how to include blood-based tests in the national cancer screening program. In the overall landscape of cancer screening, different stakeholders have different views on the matter. Patients and clinicians alike will want any patient with cancers to be diagnosed earlier, while developers and manufacturers of the test will ultimately focus on monetary returns as a primary consideration. Policy makers and government agencies have to determine the cost-effectiveness of such tests and be mindful of all the additional subsequent more invasive and expensive tests that would be performed in the presence of a positive test result, all of which can compound health care costs. Aside from the financial aspect, the other downsides of screening, such as patient anxiety and lead-time bias, will all need to be considered seriously. Most of the existing studies examining the attitude and preference for blood-based CRC tests were conducted in Western societies [26,27]. Preference of the technology in Asia is understudied. Therefore, studying preferences in Singapore, with its mix of East, South, and Southeast Asian cultures, provides a valuable addition to the literature.

Screening test only serves as the first step—follow-up diagnostic tests are required to complete the process. The gaps between stool-based tests and follow-up colonoscopies have been documented in the literature [55,56], which compromises the benefit of the screening program. Nonetheless, blood-based tests give policy makers another option to improve CRC screening. While blood-based tests in themselves may result in higher colonoscopy referral rates, blood-based tests may be combined with the existing screening methods rather than replacing them. This potentially improves on current CRC screening program by reducing the burden of colonoscopy through a 2-step screening approach that triages positive stool-based test patients [57] and serves as an alternative for people who reject stool-based tests [22]. Additional research is required to address these practical issues and understand the value brought by blood-based tests before advocating for the inclusion of blood-based cancer screening tests into the national guidelines.

### Limitations

We acknowledge several limitations in this study. First, the DCE could not encompass the entirety of decision attributes pertinent to CRC screening, potentially limiting the comprehensive representation of individual decision matrices. However, we prioritized the most salient attributes of the population through qualitative interviews. Moreover, the qualitative interviews helped furnish and provide supplementary perspectives beyond the finalized attributes. Second, it is imperative to note that our study was conducted within a relatively affluent nation, thereby limiting the generalizability of economic considerations, such as income sensitivity and trade-offs to settings with lower income levels. In addition, the availability, accessibility, and quality perception of essential infrastructure, services, and resources may be influenced by local contexts, and their differential manifestations in various settings could yield disparate research conclusions.

Nevertheless, this study advances our understanding of the preferences of the population for CRC screening tests with respect to the type of procedure. The quantified value of the population’s preferences can help in the design of more targeted policies to promote optimal screening behavior and improve screening rates. Given the constrained available resources, more resources can be allocated in the short term to (1) increase the awareness of noninvasive tests and (2) the accessibility of noninvasive tests. Given that stool-based tests are nationally recommended in Singapore, efforts addressing the emotional barriers of embarrassment and disgust of stool collection should be promoted to encourage the collection of stool as something fundamental and shameless. In the long-run, policy makers should consider investing in research and development to improve the accuracy of blood-based tests, as they are generally preferred over invasive tests and may lead to greater uptake. With an improved blood-based test that yields higher sensitivity and specificity rates, institutionalizing CRC screening alongside other routine blood works is likely to be widely acceptable.

## Appendix

Multimedia Appendix 1 Attributes and levels of the discrete choice experiment with the total number of appearances and selections.

Multimedia Appendix 2 Sociodemographic of participants from the qualitative survey and the discrete choice experiment.

Multimedia Appendix 3 Full mixed-mixed multinomial logit analysis table.

## Abbreviations

CRC: colorectal cancer
CRI: conditional relative importance
DCE: discrete choice experiment
FIT: fecal immunochemical testing
MMML: mixed-mixed multinomial logit
MXL: mixed logit
NUS: National University of Singapore
RCT: randomized controlled trial

## Data Availability

Data are available upon request, subject to the approval of the review boards.
