# Supplementary material for: Advancing Colorectal Cancer Detection With Blood-Based Tests: Qualitative Study and Discrete Choice Experiment to Elicit Population Preferences
Source: JMIR Public Health Surveill. 2024 Jul 17;10:e53200. doi: 10.2196/53200 (PMC11292146; doi:10.2196/53200)
Supplement: Multimedia Appendix 1 [file publichealth_v10i1e53200_app1.docx]

| **Supplementary Table 1. Attributes and levels of the discrete choice experiment with the total number of appearances and selections** | | | | |
| --- | --- | --- | --- | --- |
| **Attribute** | **Levels** | **Total No. of Appearance** | **Total No. of Selection** | **Percentage of Selection** |
| **Procedure** | Colonoscopy | 6159 | 3003 | 49% |
|  | CT colonography | 5380 | 2139 | 40% |
|  | Stool-based (2 Days) | 2143 | 1261 | 59% |
|  | Stool-based (1 Day) | 1998 | 1258 | 63% |
|  | Blood-based | 4740 | 2549 | 54% |
| **Pain Level** | No pain | 11419 | 6315 | 55% |
|  | Mild pain | 9001 | 3895 | 43% |
| **Sensitivity** | 100% | 6959 | 4761 | 68% |
|  | 95% | 6911 | 2835 | 41% |
|  | 80% | 4554 | 1822 | 40% |
|  | 60% | 1996 | 792 | 40% |
| **Recommendation** | Health Promotion Board | 4905 | 2872 | 59% |
|  | Doctors | 6766 | 3581 | 53% |
|  | Family or friends | 4511 | 2167 | 48% |
|  | Neither | 4238 | 1590 | 38% |
| **Cost** | Singapore $0 | 4453 | 3210 | 72% |
|  | Singapore $5 (~US $3.75) | 1594 | 1211 | 76% |
|  | Singapore $30 (~US $22.50) | 5510 | 2718 | 49% |
|  | Singapore $400 (~US $300) | 3929 | 1568 | 40% |
|  | Singapore $1000 (~US $750) | 4934 | 1503 | 31% |
| **Risk of Test** | No risk | 15282 | 8567 | 56% |
|  | 1% risk of adverse event | 5138 | 1643 | 32% |
| **Opt-Out** |  | 10210 | 4567 | 48% |
