# Supplementary material for: Advancing Colorectal Cancer Detection With Blood-Based Tests: Qualitative Study and Discrete Choice Experiment to Elicit Population Preferences
Source: JMIR Public Health Surveill. 2024 Jul 17;10:e53200. doi: 10.2196/53200 (PMC11292146; doi:10.2196/53200)
Supplement: Multimedia Appendix 2 [file publichealth_v10i1e53200_app2.docx]

| **Supplementary Table 2. Sociodemographic variable - Qualitative** | | |
| --- | --- | --- |
|  |  | **Total (n=30)** |
| **Gender** | |  |
|  | Female | 18 (60%) |
|  | Male | 12 (40%) |
| **Ethnicity** | |  |
|  | Chinese | 24 (80%) |
|  | Non-Chinese | 6 (20%) |
| **Age** |  |  |
|  | 40 years to 60 years | 15 (50%) |
|  | 61 years and above | 15 (50%) |
| **Family History of CRC** | |  |
|  | Yes | 4 (13%) |
|  | No | 26 (87%) |
| **CRC Screening History** | |  |
|  | Colonoscopy | 14 (47%) |
|  | Stool-Based Test | 20 (67%) |
|  | Blood-Based Test | 6 (20%) |
|  | None | 5 (17%) |
|  |  |  |

| **Supplementary Table 3. Sociodemographic variable - Quantitative** | | | |  |  |
| --- | --- | --- | --- | --- | --- |
|  |  | **Total**  **(n=1062)** | **Passed Fixed Tasks**  **(n=1021)** | **Failed Fixed Tasks**  **(n=41)** | **p-value** |
| **Gender** | |  |  |  |  |
|  | Female | 608 (57%) | 588 (58%) | 20 (49%) | 0.26 |
|  | Male | 454 (43%) | 433 (42%) | 21 (51%) |  |
| **Ethnicity** | |  |  |  |  |
|  | Chinese | 909 (86%) | 874 (86%) | 35 (85%) | 0.97 |
|  | Non-Chinese | 153 (14%) | 147 (14%) | 6 (15%) |  |
| **Age** |  |  |  |  |  |
|  | 40 years to 60 years | 665 (63%) | 640 (63%) | 25 (61%) | 0.83 |
|  | 61 years and above | 397(37%) | 381 (37%) | 16 (39%) |  |
| **Household Income Level** | |  |  |  |  |
|  | High Income ($6,000 and above) | 462 (44%) | 448 (44%) | 14 (34%) | 0.22 |
|  | Lower Income ($5,999 and below) | 600 (57%) | 573 (56%) | 27 (66%) |  |
| **Marital Status** | |  |  |  |  |
|  | Married | 770 (73%) | 742 (73%) | 28 (68%) | 0.54 |
|  | Single/Divorced/Widowed/Separated | 292 (28%) | 279 (27%) | 13 (32%) |  |
| **Education Level** | |  |  |  |  |
|  | Primary & Secondary | 269 (25%) | 258 (25%) | 11 (27%) | 0.12 |
|  | Pre-University | 355 (33%) | 336 (33%) | 19 (46%) |  |
|  | University and above | 438 (41%) | 427 (42%) | 11 (27%) |  |
| **Housing Type** | |  |  |  |  |
|  | Public Housing | 936 (88%) | 898 (88%) | 38 (93%) | 0.36 |
|  | Private Housing | 126 (12%) | 123 (12%) | 3 (7%) |  |
| **Working Status** | |  |  |  |  |
|  | Currently working | 781 (74%) | 750 (74%) | 31 (76%) | 0.76 |
|  | Not working/retired/student | 281 (27%) | 271 (27%) | 10 (24%) |  |
| **Family History of CRC** | |  |  |  |  |
|  | Yes | 127 (12%) | 125 (12%) | 2 (5%) | 0.15 |
|  | No | 935 (88%) | 896 (88%) | 39 (95%) |  |
| **CRC Screening History** | |  |  |  |  |
|  | Yes | 454 (43%) | 435 (43%) | 19 (46%) | 0.64 |
|  | No | 608 (57%) | 586 (57%) | 22 (54%) |  |
| **Perceived Risk of Test Score** | | 14.48 (2.9) | 14.51 (2.9) | 13.76 (2.6) | 0.29 |
| **Social Support Score** | | 19.23 (4.2) | 19.24 (4.2) | 19.12 (4.2) | 0.90 |
| **Present Orientation** | | 12.72 (3.8) | 12.67 (3.8) | 14.02 (4.1) | 0.01 |
| **Intolerance of Uncertainty** | | 32.98 (8.8) | 32.97 (8.7) | 33.07 (10.3) | 0.34 |
| **Note: Sociodemographic variables are presented as absolute numbers and corresponding proportions in percentages for categorical variables such as gender. For continuous variables like scores, the mean score along with the standard deviation is provided in brackets for reference.** | | | | | |
