# Supplementary material for: Advancing Colorectal Cancer Detection With Blood-Based Tests: Qualitative Study and Discrete Choice Experiment to Elicit Population Preferences
Source: JMIR Public Health Surveill. 2024 Jul 17;10:e53200. doi: 10.2196/53200 (PMC11292146; doi:10.2196/53200)
Supplement: Multimedia Appendix 3 [file publichealth_v10i1e53200_app3.docx]

| **Supplementary Table 4. Full mixed–mixed multinomial logit analysis^a^** | | | | | | | |
| --- | --- | --- | --- | --- | --- | --- | --- |
|  |  | **Class 1: Strong supporters** | | | **Class 2: Weak supporter** | | |
|  |  | **Coefficient** | **95% CI** | **P value** | **Coefficient** | **95% CI** | **P value** |
| **Mean Coefficient** | |  |  |  |  |  |  |
| **Left** |  | 0.28 | 0.15 to 0.42 | <0.001 | 0.08 | –0.02 to 0.18 | .13 |
| **None** | | −5.13 | –5.79 to –4.46 | <0.001 | −0.86 | –1.10 to –0.61 | <0.001 |
| **Procedure** | |  |  |  |  |  |  |
|  | Colonoscopy | −0.35 | –0.71 to 0.02 | 0.07 | −1.05 | –1.28 to –0.81 | <0.001 |
|  | CT^b^ colonography | −0.53 | –0.87 to –0.18 | <0.001 | −1.01 | –1.23 to –0.78 | <0.001 |
|  | Stool-based (2 Days) | 0.00 | Reference | ­–^c^ | 0.00 | Reference | – |
|  | Stool-based (1 Day) | −0.20 | –0.57 to 0.16 | 0.27 | 0.63 | 0.39 to 0.87 | <0.001 |
|  | Blood–based | 0.06 | –0.27 to 0.49 | 0.70 | 0.66 | 0.44 to 0.88 | <0.001 |
| **Pain level** | |  |  |  |  |  |  |
|  | No pain | 0.00 | Reference | – | 0.00 | Reference | – |
|  | Mild pain | −0.25 | –0.46 to –0.04 | 0.02 | −0.84 | –0.98 to –0.70 | <0.001 |
| **Sensitivity** | |  |  |  |  |  |  |
|  | 100% | 2.92 | 2.61 to 3.24 | <0.001 | 1.25 | 1.10 to 1.41 | <0.001 |
|  | 95% | 1.58 | 1.29 to 1.87 | <0.001 | 0.42 | 0.25 to 0.58 | <0.001 |
|  | 80% | 0.00 | Reference | – | 0.00 | Reference | – |
|  | 60% | −1.65 | –2.04 to –1.27 | <0.001 | −0.96 | –1.19 to –0.72 | <0.001 |
| **Recommendation** | |  |  |  |  |  |  |
|  | Health Promotion Board | 1.24 | 0.99 to 1.49 | <0.001 | 0.79 | 0.62 to 0.96 | <0.001 |
|  | Doctors | 0.89 | 0.64 to 1.14 | <0.001 | 0.67 | 0.50 to 0.83 | <0.001 |
|  | Family & Friend | 0.17 | –0.08 to 0.43 | 0.18 | 0.40 | 0.22 to 0.58 | <0.001 |
|  | Neither | 0.00 | Reference | – | 0.00 | Reference | – |
| **Cost** | |  |  |  |  |  |  |
|  | Singapore $0 | 0.00 | Reference | – | 0.00 | Reference | – |
|  | Singapore $5 | −0.67 | –1.02 to –0.32 | <0.001 | −0.33 | –0.55 to –0.11 | <0.001 |
|  | Singapore $30 | −0.97 | –1.24 to –0.70 | <0.001 | −0.83 | –0.99 to –0.66 | <0.001 |
|  | Singapore $400 | −2.24 | –2.55 to –1.94 | <0.001 | −2.93 | –3.14 to –2.72 | <0.001 |
|  | Singapore $1000 | −4.00 | –4.41 to –3.59 | <0.001 | −4.46 | –4.75 to –4.18 | <0.001 |
| **Risk of test** | |  |  |  |  |  |  |
|  | No risk | 0.00 | Reference | – | 0.00 | Reference | – |
|  | 1% risk of adverse event | −0.60 | –0.85 to –0.35 | <0.001 | −1.04 | –1.21 to –0.87 | <0.001 |
| **Standard Deviation** | |  |  |  |  |  |  |
| **None** | | 5.46 | 4.85 to 6.07 | <0.001 | 2.87 | 2.69 to 3.06 | <0.001 |
| **Procedure** | |  |  |  |  |  |  |
|  | Colonoscopy | 0.68 | 0.40 to 0.96 | <0.001 | 1.05 | 0.86 to 1.23 | <0.001 |
|  | CT colonography | 1.09 | 0.81 to 1.37 | <0.001 | 0.45 | 0.24 to 0.66 | <0.001 |
|  | Stool-based (2 Days) | 0.00 | Reference | – | 0.00 | Reference | – |
|  | Stool-based (1 Day) | 0.44 | –0.01 to 0.89 | 0.06 | 0.62 | 0.30 to 0.94 | <0.001 |
|  | Blood–based | 0.46 | 0.16 to 0.75 | <0.001 | 1.10 | 0.92 to 1.28 | <0.001 |
| **Pain level** | |  |  |  |  |  |  |
|  | No pain | 0.00 | Reference | – | 0.00 | Reference | – |
|  | Mild pain | 0.04 | –0.22 to 0.29 | 0.79 | 0.48 | 0.31 to 0.64 | <0.001 |
| **Sensitivity** | |  |  |  |  |  |  |
|  | 100% | 0.46 | 0.19 to 0.73 | <0.001 | 0.30 | 0.12 to 0.48 | <0.001 |
|  | 95% | 0.01 | –0.30 to 0.32 | 0.96 | 0.18 | –0.01 to 0.38 | <0.001 |
|  | 80% | 0.00 | Reference | – | 0.00 | Reference | – |
|  | 60% | 0.98 | 0.49 to 1.46 | <0.001 | 1.59 | 1.35 to 1.83 | <0.001 |
| **Recommendation** | |  |  |  |  |  |  |
|  | Health Promotion Board | 0.50 | 0.24 to 0.77 | <0.001 | 0.07 | –0.10 to 0.25 | .40 |
|  | Doctors | 0.60 | 0.32 to 0.89 | <0.001 | 0.08 | –0.11 to 0.27 | .43 |
|  | Family & friend | 0.10 | –0.18 to 0.37 | 0.50 | 0.19 | –0.01 to 0.39 | .07 |
|  | Neither | 0.00 | Reference | – | 0.00 | Reference | – |
| **Cost** | |  |  |  |  |  |  |
|  | Singapore $0 | 0.00 | Reference | – | 0.00 | Reference | – |
|  | Singapore $5 | 1.25 | 0.86 to 1.63 | <0.001 | 0.14 | –0.15 to 0.42 | .35 |
|  | Singapore $30 | 0.13 | –0.15 to 0.42 | 0.36 | 0.02 | –0.18 to 0.22 | .86 |
|  | Singapore $400 | 0.68 | 0.38 to 0.98 | <0.001 | 0.62 | 0.43 to 0.82 | <0.001 |
|  | Singapore $1000 | 2.08 | 1.73 to 2.43 | <0.001 | 1.37 | 1.11 to 1.63 | <0.001 |
| **Risk of Test** | |  |  |  |  |  |  |
|  | No risk | 0.00 | Reference | – | 0.00 | Reference | – |
|  | 1% risk of adverse event | 0.28 | –0.01 to 0.56 | 0.06 | 0.26 | 0.05 to 0.47 | <0.001 |
| **Class membership** | |  |  |  |  |  |  |
| **Sex** | |  |  |  |  |  |  |
|  | Female |  |  |  | 0.19 | 0.11 to 0.27 | <0.001 |
|  | Male |  |  |  | 0.00 | Reference | – |
| **Ethnicity** | |  |  |  |  |  |  |
|  | Chinese |  |  |  | 0.52 | 0.41 to 0.63 | <0.001 |
|  | Non–Chinese |  |  |  | 0.00 | Reference | – |
| **Age** |  |  |  |  |  |  |  |
|  | 40 years to 60 years |  |  |  | 0.00 | Reference | – |
|  | 61 years and above |  |  |  | −0.52 | –0.61 to –0.42 | <0.001 |
| **Household income level Singapore $** | |  |  |  |  |  |  |
|  | High Income (>6000) |  |  |  | −0.13 | –0.22 to –0.05 | <0.001 |
|  | Lower Income (≤5999) |  |  |  | 0.00 | Reference | – |
| **Marital status** | |  |  |  |  |  |  |
|  | Married |  |  |  | −0.06 | –0.16 to 0.04 | .21 |
|  | Single or divorced or widowed or separated |  |  |  | 0.00 | Reference | – |
| **Education Level** | |  |  |  |  |  |  |
|  | Primary & secondary |  |  |  | 0.08 | –0.02 to 0.19 | .13 |
|  | Pre–university |  |  |  | 0.00 | Reference | – |
|  | University and above |  |  |  | 0.02 | –0.22 to –0.05 | .61 |
| **Housing Type** | |  |  |  |  |  |  |
|  | Public housing |  |  |  | 0.00 | Reference | – |
|  | Private housing |  |  |  | −0.47 | –0.60 to –0.35 | <0.001 |
| **Working Status** | |  |  |  |  |  |  |
|  | Currently working |  |  |  | −0.24 | –0.34 to –0.14 | <0.001 |
|  | Not working or retired or student |  |  |  | 0.00 | Reference | – |
| **Family history of CRC^d^** | |  |  |  |  |  |  |
|  | Yes |  |  |  | −0.74 | –0.85 to –0.63 | <0.001 |
|  | No |  |  |  | 0.00 | Reference | – |
| **CRC screening history** | |  |  |  |  |  |  |
|  | Yes |  |  |  | −0.52 | –0.60 to –0.44 | <0.001 |
|  | No |  |  |  | 0.00 | Reference | – |
| **Perceived safety of test Score** | |  |  |  | −0.16 | –0.17 to –0.14 | <0.001 |
| **Social support score** | |  |  |  | −0.03 | –0.05 to –0.02 | <0.001 |
| **Present orientation** | |  |  |  | −0.03 | –0.05 to –0.02 | <0.001 |
| **Intolerance of uncertainty** | |  |  |  | −0.01 | –0.02 to –0.01 | <0.001 |
| Log–likelihood | | −8041 |  |  |  |  |  |
| AIC^e^ |  | 16257 |  |  |  |  |  |
| BIC^f^ |  | 16955 |  |  |  |  |  |
| ^a^Mean coefficient refers to the population mean. Standard deviation measures the individual preference heterogeneity. A significant value means that the preference for the corresponding level is heterogeneous at the individual levels.  ^b^CT: computed tomography.  ^c^Not applicable.  ^d^CRC: colorectal cancer.  ^e^AIC: Akaike information criteria.  ^f^BIC: Bayesian information criteria. | | | | | | | |
